# Impact of the SUture BIte TEchnique on clinical outcomes after midline laparotomy closure: SUBITE—a systematic review and meta-analysis

**DOI:** 10.1007/s10029-026-03700-z

**Published:** 2026-05-19

**Authors:** Markus Golling, Petra Baumann, Fabian Kuger, René H. Fortelny

**Affiliations:** 1 Sigmund Freud Private University Vienna: Sigmund Freud PrivatUniversitat Wien, Vienna, Austria; 2 DIAK Hospital, County Hospital Schwaebisch Hall, Department of General & Visceral Surgery, Diakoniestrasse 10, Schwaebisch Hall, 75432 Germany; 3 Department of Medical Scientific Affairs, Aesculap AG, Tuttlingen, Am Aesculap Platz 78532 Germany

**Keywords:** Midline laparotomy, Abdominal wall closure, Small bites, Large bites, Suture technique, Incisional hernia, Surgical site infections, Fascial dehiscence, Meta-analysis, Systematic review

## Abstract

**Background:**

The objective of this Systematic Review and Meta-Analysis (SR/MA) was to identify the best suture technique (short or large bites) for abdominal wall closure with respect to relevant outcome parameters such as incisional hernia (IH), surgical site infection (SSI) and linea alba (aponeurotic layer of the abdominal wall) dehiscence (LAD).

**Methods:**

Registration was done in PROSPERO, a systematic literature search was performed in three data bases (PubMed, Embase and Cochrane). Randomised controlled (RCT) as well as non-randomised controlled trials (n-RCT) comparing the short (SB) versus large bite (LB) technique for abdominal wall closure after midline laparotomy were eligible for inclusion. Quality assessment was performed for RCTs (ROB) & n-RCTs (ROBINS-1, MINORS). The incidence of IH, SSI, LAD as well as the length of hospital stay (LOS) and time to close the linea alba (aponeurotic layer of the abdominal wall) were analysed as outcome parameters. Odds ratio with 95% confidence intervals were chosen to determine statistical significance. Heterogeneity was explored using the I^2^-statistics and funnel plots evaluated a possible publication bias.

**Results:**

This SR/MA comprised in total 5886 patients (large bite group 3339 vs. 2547 short bite group) enrolled in 7 RCTs and 5 n-RCTs. The SB-technique was associated with a significant lower IH, SSI, FD rate and a shorter LOS compared to the LB-technique (IH: Odds Ratio OR = 0.47 (95% CI 0.38–0.58; *p* < 0.00001; I^2^ = 11%)); SSI: OR = 0.53 (95% CI 0.42–0.67; *p* < 0.00001; I^2^ = 0%); FD: OR = 0.60 (95% CI 0.38–0.93; *p* = 0.02; I^2^ = 0%); LOS: Mean difference in days MD = -1.04 (95% CI -1.70, -0.37; *p* = 0.002; I^2^ = 13%), respectively). Furthermore, pooled effect estimates derived from RCTs were comparable to n-RCTs. No statistical relevant publication bias was detected, and the confidence of resulting evidence was high according to the validated GRADE tool.

**Conclusion:**

This systematic review and meta-analysis demonstrate consistent reductions in incisional hernia and surgical site infection with the small bites technique for midline laparotomy closure. The direction of effect is stable across randomized and comparative studies and supported by available long-term data. The clinical relevance and consistency of these findings support preferential use of the small bites technique in routine practice. The present evidence provides a robust basis for consideration in future updates of EHS abdominal wall closure guidelines.

**Registration:**

PROSPERO, registration number: CRD420251033244, registration date: 16th April 2025.

**Supplementary Information:**

The online version contains supplementary material available at 10.1007/s10029-026-03700-z.

## Introduction

### Rationale

The midline laparotomy, either performed in an elective or emergency setting is the most common procedure to get access to the abdominal cavity [1]. Postoperative complications have been reported occurring in the short-term course such as linea alba dehiscence (burst abdomen + wound dehiscence) and surgical site infections (SSI), as well as in the long-term course, like incisional hernias (IH). They are mentioned in 1–3% (FD), up to 25% (SSI, depending on the surgical intervention) and up to 20% (IH, until 1 year postoperatively). Special risk factors like BMI > 25, abdominal aortic aneurysms (AAA), high age, diabetes mellitus, colorectal or contaminated surgery predispose to even higher rates [2]. Moreover, the rate of IH is time line dependent [3].

Numerous clinical studies have been performed to analyse the effect of the suture material (braided vs. monofilament, absorbable vs. non-absorbable) and the suture technique (continuous vs. interrupted) applied to close the midline after surgery. The INLINE Meta-Analysis published in 2010 has shown that the combination of a long-term absorbable monofilament suture material applied in the continuous suture technique leads to a lower complication rate following elective midline laparotomies [4]. Nevertheless, rates of SSI and IH remained high. Since then, further concepts of elastic extra-long term resorbable [5] and antimicrobial-coated suture materials (Triclosan, Chlorhexidine) were introduced by the industry while technical efforts (> 4:1 suture/wound length ratio, short stitch) were suggested by surgical teams (Stitch-trial, ESTOIH-study) to lower FD, SSI and IH.

In 1997, Israelsson was the first, who compared SB vs. LB to close midline laparotomies [6]. More evidence on this topic was provided in further randomised studies thereafter [7–9].

Currently, there are 8 Meta-Analysis and Systematic Reviews (MA/SR) available focusing on the suture stitch technique for abdominal wall closure, which differ in content and scientific questioning (PICO model) [10–17]. Two SR/MA are based either on old literature or included only a small number of studies [10, 11], two have compared different modified suture techniques for midline closure [12, 13]. More recent SR/MA published in 2023 and 2025 analysed the literature available until 2021 or 2024 and focus only on the outcome of RCTs [14, 15]. The SR/MAs performed by Rassador et al. and Morarascu et al. analysed the literature published until May 2023 or Nov 2024 and included RCTs (*n* = 3 or *n* = 4 respectively) as well as n-RCTs (*n* = 3 each) [16, 17].

### Objectives

The present SR/MA summarises the literature comparing the SB versus LB-technique published until the beginning of February 2025. We presumed, that the inclusion of prospective, comparative n-RCT may increase the strength of a Meta-Analysis [18]. Thus, in contrast to Yii et al. and Alrashidi et al., the aim of our current Meta-Analysis is to include RCTs and n-RCTs (comparative cohort studies) to provide evidence facilitating health care decisions. It was shown by Abraham et al. that the result and strength of a SR/MA based on RCTs only were comparable to those including only well-designed n-RCTs [19].

The current strength of the EHS (European Hernia Society) recommendation regarding the suture stitch technique for abdominal wall closure is low and solely based on the outcome of 2 RCTs [20, 21]. It is our expectation for the present SR/MA that a higher clinical evidence level can be generated, serving as a basis for an update on the suture technique for midline laparotomy closures.

## Methods

### Protocol and registration

A-priori study protocol according to PRISMA-P (Preferred Reporting Items for Systematic reviews and Meta-Analysis Protocols), was prepared in advance of the final conduct of the SR/MA for clarity, transparency and future reproducibility [22, 23].

To fulfill current requirements for SR/MA, our a priori developed PRISMA protocol was proactively registered and published in the international, prospective register of ongoing systematic reviews (PROSPERO, http://www.crd.york.ac.uk/prospero/) on the 16th April 2025 under registration number CRD420251032244 (PROSPERO).

As a guidance for the reporting of the present SR/MA the PRISMA statement was applied [24, 25].

### Eligibility criteria

To structure the research question, the PICOTS (Population, Intervention, Comparison, Outcome, Time, Setting) format/model was used. Studies that fulfilled the inclusion criteria are shown in Table 1. Exclusion criterias were: (1) modified closure techniques for laparotomy closure, (2) the usage of a prophylactic mesh, (3) systematic reviews, meta-analysis, case reports, case series, study protocols, surveys, letters, comments, guidelines, biomechanical study, animal study, and (4) unpublished data or material.

**Table 1:** PICOTS model of SUBITE SR/MA

Structure	Meaning	Inclusion criteria
P	Population /Patients	Adult patients (age≥18 years)
I	Intervention	Electice as well as emergent median laparotomy or relaparotomy
C	Comparison	Short bite/stitch versus long bite/stitch suture techniquefor abdominal wall closure
O	Outcome	Primary: Incisional herniaSecondary: SSI, FD, LOS, Closure time, SL/WL ratio
T	Time	No limit regarding follow-up duration
S	Setting	RCT as well as comparative non-RCTNo limit regarding sample size and type of languageNo restriction to geographical location of the clinics participating in the studiesNot all outcomes of interest need to be reported in a studyPublication until end of January 2025

Legend: *LAD* Linea Alba Dehiscence, *LOS* Length of Hospital Stay, *MA* Meta-Analysis, *RCT* Randomised Controlled Trial, *SSI* Surgical Site Infection, *SR* Systematic Review

#### Outcome parameters

- *Primary outcome parameter* was the incisional hernia (IH) rate occurring at least within 6 months after surgery diagnosed either by physical examination, ultrasound or radiological examination (CT or MRI) as defined in each study. Alternatively, the EHS definition `any abdominal wall gap with or without a bulge around a postoperative scar perceptible or palpable by clinical examination or imaging` [17].
- *Secondary parameters* were surgical site infections (SSI), linea alba dehiscence (LAD) including secondary wound dehiscence & burst abdomen, length of postoperative stay (LOS), suture length to wound length ratio (SL: WL), and the time to close the midline.

Regarding SSI, only superficial and deep surgical site infections classified as A1 and A2 according to the Centre for Disease Control and Prevention (CDC) were included and analysed (https://www.cdc.gov/surgical-site-infections/about/index.html). SSI-studies should have a follow up time of at least 30 days.

LAD was defined as a clinically evident rupture of the laparotomy wound until 10 days postoperatively with the need of an emergency reoperation [7]. LAD thus incorporates `burst abdomen` as well as `secondary wound dehiscence`, both commonly used interchangeably and not clearly distinguished in the clinical setting. Duration of follow-up should be a least 1 month. The LOS was the duration from day of surgery until day of discharge and was used as mentioned in individual studies. Closure time and SL: WL ratio was used as reported in individual studies.

### Information sources and Search strategy

A well-organized literature search was done by the involvement of a search specialist using various medical subject headings (MESH) and keywords in different databases in combination with Boolean operators to ensure a high-quality search strategy. The MESH terms and keywords used for the selection of eligible literature were developed by two persons with expertise in the field of abdominal wall closure (PB and RHF). Information sources were bibliographic data bases such as PubMed, EMBASE and Cochrane. The electronic database search was supplemented by the search for trial protocols in clinical trials registries such as clinicaltrials.gov. To ensure literature saturation, the reference list of the included articles was checked in addition. The search was performed on January 10th, 2025. The detailed literature search strategies for each individual data base including customization based on specific attributes are presented in the supplement (Supplement: S1 (PubMed), S2 (EMBASE), S3 (Cochrane)).

### Selection process

The Citavi tool (https://www.citavi.com) was used to upload the literature search results and to deduplicate references during the review process. A bibliography of the result was developed and provided to the research team. Two independent assessors (PB & RHF) screened the title and abstract of the articles yield by the literature search against the inclusion criteria to determine eligibility. The result was discussed, and disagreements were solved by the involvement of a third assessor (MG) for decision and consensus. The reference details of the considered and selected articles were listed in a reference tool (Citavi) and the full-text pdfs were ordered at a library. The full-text pdfs were checked whether these meet the inclusion criteria and predefined outcomes. Evaluation was done by two independent assessors (PB and RHF), disagreements were resolved by discussion with a third evaluator (MG). For articles which were excluded, the reason was recorded. No blinding was made to the authors or journal titles of the articles/studies.

### Data collection process and data items

Pre-defined and selected variables and outcomes were extracted from the eligible studies by one researcher (PB) and tabular listed in an excel sheet for final inclusion decision. Outcomes were collected and extracted as reported in individual studies. To reduce bias and errors, the extracted data were verified and confirmed by two other independent assessors (RHF and MG). Discrepancies were resolved by discussion to arrive at a final decision and agreement.

The information and variables were extracted from the eligible articles and tabulated and synthesised in a structured excel sheet. The characteristics data of each selected study is shown in the `overview table`(Table 2), which includes the following details: name of the first author, quality assessment according to Scottish Intercollegiate Guideline Network (SIGN), year of publication, recruitment period, total number of patients and per group, study design (RCT or n-RCT), number of participating clinics/departments, name of the country in which the study was performed, type of laparotomy (elective, emergency or relaparotomy), interventional group (short or long stitch technique), suture material details, follow-up period, SL: WL ratio, details regarding the stitch to stitch distance (stitch interval), the distance from the incision to the lateral wound edge (stitch width) and the classical outcome parameters (incisional hernias (IH), surgical site infection (SSI) & linea alba dehiscence (LAD).

**Table 2:** Overview of the baseline characteristics of RCTs and n-RCTs

Author	SIGN Quality Assessment	Year	Recruitment period	Number of patients	Study design	Number of centres	Country	Type of laparotomy
Millbourn [7]	Acceptable	2009	Jan 2011 - Jan 2006	737(SB: 381 / LB: 356)	RCT	1	Sweden	Elective and emergencymidline laparotomy
Deerenberg [8]	High	2015	Oct 2009- Marc 2012	560(SB:284 / LB:276 )	RCT	10	Netherlands	Elective midline laparotomy
Albertsmeier / Fortelny [9, 26]	High	2021, 2022	Mar 2014- Dec 2019	425(SB:215 / LB: 210)	RCT	9	Germany, Austria	Elective midline laparotomy
Lai [27]	High	2020	Feb 2017- Sept. 2018	86(SB: 42/ LB: 44)	RCT	2	Malaysia	Elective midline laparotomy
Probst [28]	High	2020	Sept 2017 - Apr 2018	100(SB:50 / LB:50)	RCT	1	Germany	Elective relaparotomy
Oczean [29]	Acceptable	2024	Jun 2019- Jun 2022	173(SB:87 / LB:86)	RCT	1	Turkey	Elective and emergency midline laparotomy
Mustaqrasool [30]	Acceptable	2020	July 2017- Oct 2018	230(SB:115 / LB:115)	RCT	1	India	Midline laparotomy
Sharma [31]	Low	2020	Sept 2017- Dec 2019		RCT	1	India	Elective midline laparotomy
Kumar [32]	Low	2016	Jan 2014-July 2015	60(SB:30 / LB:30)	RCT	2	India	Elective midline laparotomy
Ain [33]	Low	2024	Jan 2023-July 2023	210SB:105 / LB:105)	RCT	2	Pakistan	Elective midline laparotomy
de Vries [34]	High	2019	Jun 2013 - Jun 2016	327(SB:136 / LB:191)	n-RCT	1	Netherlands	Elective and emergency midline laparotomy
Ghai [35]	High	2023	Jan 2021 - Dec 2021	90SB: 45 / LB:45)	n-RCT	1	India	Elective midline laparotomies
Hassan [36]	Low	2018	2015-2018		n-RCT	1	India	Elective and emergency midline laparotomy
Tolstrup [37]	Acceptable	2017	May 2009-May 2013 and Jun 2014- Oct 2015	1573(LB: 1079 / SB: 494)	n-RCT	1	Denmark	Emergency midline laparotomy
Söderbäck [38]	High	2022	2010-2011 and 2016-2017	1120(LB: 602 / SB: 518)	n-RCT	1	Sweden	Emergency and elective midline laparotomy
Thorup [39]	Acceptable	2019	May 2009-May 2013 and Jun 2014- Oct 2015	465 (SB:180 / LB: 285)	n-RCT	1	Denmark	Emergency midline laparotomy
Author	Closure method	Intervention(Suture material details)	Comparison(suture material details)	Postoperative follow-up	SL:WL ratio	Small bite technique details (stitch intervall and distance from the incison to the wound edge)	Large bite technique details(stitch intervall and distance from the incision to the wound edge)	Outcome parameters
Millbourn [7]	SB: only fasciaLB: previous standard technique	PDS II USP 2/0, 20mm needle	PDS II USP 1-0, 41 mm needle	1 year	SB > 4:1LB > 4:1	<5mm and 5mm	<5mm and 1 cm	Wound dehiscence, Surgical site infection, Incisional hernia
Deerenberg [8]	SB: only fasciaLB: mass closure	PDS Plus II, USP 2/0, 31mm needle	PDS Plus II, USP 1 loop, 48mm needle	1 year	SB ≥4:1LB ≥4:1	5mm and 5mm	1 cm and 1 cm	Incisional hernia, Surgical site infection, Burst abdomen (Fascia dehiscence)
Albertsmeier / Fortelny [9, 26]	SB: only fasciaLB: only fascia	Monomax USP2/0, 26mm needle	Monomax USP 1 loop, 48mm needle	1, 3, 5 years	SB > 5:1LB 4:1	5mm and 5 -8 mm	1 cm and 1 cm	Incisonal hernia, Surgical site infection, Burst abdomen
Lai [27]		Monomax USP2/0, 26mm needle	Monomax USP1 loop, 48 needle	8 weeks	SB 5.3:1LB 4.2:1	5mm and 5mm	1 cm and 1 cm	Surgical site infections, Burst abdomen
Probst [28]	SB: NALB: NA	Monomax USP 2/0	PDS II USP1 loop	1 year	NA	2-5mm and 5mm	15-20mm and 10mm	Incisional hernia, Surgical site infection, Burst abdomen
Oczean [29]		PDS II Plus No1, 48mm needle	PDS II Plus No1, 48mm needle	12 months	SB 5.2LB 3.6	5mm and 5 mm	1cm and 1cm	Surgical site infection, Incisional hernia,
Mustaqrasool [30]		Ethilon No.1	Ethilon No1	12 months	SB ≥ 4:1LB ≥4:1	5mm and 5mm	1 cm and 1 cm	Incisional hernia, Surgical site infection, Wound dehiscence
Sharma [31]		PDS II No 1	PDS II No 1	6 months	SB 5:1LB 4:1	5mm and 5mm	1 cm and 1 cm	Wound infection, Wound dehiscence, Incisional hernia
Kumar [32]		Ethilon loop	Ethilon loop	NA	SB ≥4:1LB: 4:1	NA and 5-8mm	NA and ≥10mm	Wound infection, Wound dehiscence, Incisional hernia
Ain [33]		Ethilon loop	Ethilon loop	NA	SB: 5.2LB: 6.4	NA	NA	Wound infection, Wound dehiscence, Incisional hernia,
de Vries [34]		PDS II, USP2/0, 31mm needle	PDS II, USP 1 loop, 48mm needle	up to 36 months	SB 4.1, NA	5mm and 5mm	1cm and 1 cm	Incisional hernia, surgical site infection, Burst abdomen
Ghai [35]	SB: only fasciaLB: NA	PDS II Plus 2/0	PDS II Plus USP 2/0	6 months		5mm and 5 mm	1cm and 1 cm	Surgical site infection, Wound dehiscence, Incisional hernia
Hassan [36]		Polypropylene No 1	Polypropylene No 1	NA		5-7mm and 5-7mm	>1cm and >1cm	Surgical site infection, Wound dehiscence
Tolstrup [37]	SB: only fasciaLB: surgeon´s decision	PDS 2/0, 36mm needle	diverse (Vicryl, PDS, Prolene)	90 days	SB ≥ 4:1LB : NA	NA	NA	Fascial dehiscence
Söderbäck [38]		PDS 2/0, small needle	PDS 0 loop, large needle	36 months	SB ≥ 4:1LB: NA	5mm and 5mm	NA	Wound dehiscence, Incisional hernia
Thorup [39]		PDS 2/0, 36mm needle	diverse (Vicryl, PDS, Prolene)	median 52 monthsmedian 19 months		NA	NA	Incisional hernia

Legend: *LB* Large Bites, *NA* not available, *n-RCT* non-Randomised Controlled Trials, *RCT* Randomised Controlled Trials, *SB* Small bites, *SL* Suture length, *WL* Wound Length

A table synchronising parameter and outcome criteria were created for selected study and suture stitch group and used for statistical analysis (Name of the author, year of publication, incidence of IH, SSI, FD, length of hospital stay (LOS, days ± SD), name of suture material, SL: WL ratio, closure time (in minutes ± SD); (data not shown).

### Study risk of bias assessment

Methodological bias was assessed by the Cochrane Collaboration tool RoB for RCTs [40, 41], the ROBINS-I tool was chosen for comparative n-RCTs [42]. In addition, the methodological quality of both study designs was evaluated by using a critical appraisal checklist developed by the Scottish Intercollegiate Guideline Network (SIGN) available for RCTs as well as for cohort studies (http://www.signacuk/methodology/indexhtml). Furthermore, the quality of n-RCTs was assessed by using the Methodological Index for n-RCTs MINORS [43]. Critical appraisal of the studies was performed by two independent assessors (PB & RHF) and disagreements were solved by discussion and conciliation with participation of a third independent assessor (MG), which is in alignment with current available guidelines [20, 21].

### Synthesis and effect measures

Studies which were sufficiently homogenous in terms of design and comparator and assessed with a `high` or `acceptable` quality using the checklist of SIGN were quantitative synthesised. Comparative Meta-Analysis of dichotomous outcome data from RCT and n-RCT were performed using weight odds ratio (OR) with 95% confidence intervals (95% CI) calculated with the Wald method. Continuous outcomes were analysed using weighted mean differences with 95% confidence intervals. Illustration of the results were done by forest plots. The forest plots showed the result depending on a specific outcome measure (e.g. IH, SSI, FD) separately for RCT and n-RCT as well as for the combination of both study designs (total). Both random and fixed effects models were tested. Fixed effects model assumed that the treatment effect in each study was identical, whereas the random effects model assumed that variations were presented between the studies.

Heterogeneity was explored using Chi^2^ test (significance level: 0.1) and I^2 −^statistics to estimate the percentage of total variation across studies, owing to heterogeneity rather than chance. I^2^ values of 0% to 40% implicated low heterogeneity (not important), 30%-60% represented moderate heterogeneity; 50–90% indicated substantial heterogeneity and 75%-100% assumed considerable heterogeneity. If high levels of heterogeneity among the studies existed (I^2^ ≥ 50% or *p* < 0.1) the study design in the included studies was analysed. Subgroup analysis was performed to explore possible source of heterogeneity based on the following criteria: sample size of the study, number of centres (mono- vs. multicentric), type of suture material. Sensitivity analysis depending on study design (RCT vs. n-RCT) and risk of bias was done. Studies with a high risk of bias were only included, when they provided the available information or critical outcome of interest. X^2^ tests were applied to assess the heterogeneity between the studies. For dichotomous data, the Mantel-Haenszel (M-H) method was used for fixed effects model if tests of heterogeneity were not significant. If statistical heterogeneity was recognized (I^2^ ≥ 50% or *p* < 0.1), the random effects model was chosen and the inverse variance (IV) method applied. For continuous data, the inverse variance method was used in either fixed effect or random effects model. If heterogeneity was substantial, a qualitative summary was performed. All p-values were two-sided. Missing data were analysed as such. For statistical analysis the RevMan Version 5.4. was used. A licence for usage was obtained from the Cochrane Collaboration, Freiburg, Germany.

Qualitative synthesis was provided in text and tables to summarise and explain the characteristics and findings of the included studies (e.g. tables which showed the characteristics and overview of the included studies or result of MINORS and RoB, ROBINS-I, GRADE assessment). This was done to explore the findings & relationship both within and between the studies.

### Reporting bias assessment

We checked if a study protocol (mainly for RCTs) was published before the recruitment started. If available, the outcomes described in the protocol were compared with the final report. When a study protocol was not published, the outcomes reported in the materials and method section were compared with the section part of the final article. In addition, we screened *clinicaltrials.gov*, if the studies included were registered & assessed selective reporting of outcome measures. In addition, fixed and random effects models were compared to evaluate a small sample size bias. If present, the random effect estimate was more beneficial than the fixed effect estimate. On top, Egger´s test evaluated the effect of small sample size studies while Funnel plots depict a potential publication /reporting bias, if ≥ 10 studies were synthesised.

### Certainty assessment

To summarise the confidence of the resulting body of evidence the established and validated tool - Grading of Recommendations Assessment, Development and Evaluation (GRADE) was used for the present review [44, 45]. The following domains were judged for quality of evidence (limitations, inconsistency, indirectness, imprecision, and publication bias). Quality was rated as high, moderate, low or very low.

## Results

### Search result and study selection

**Fig. 1:**
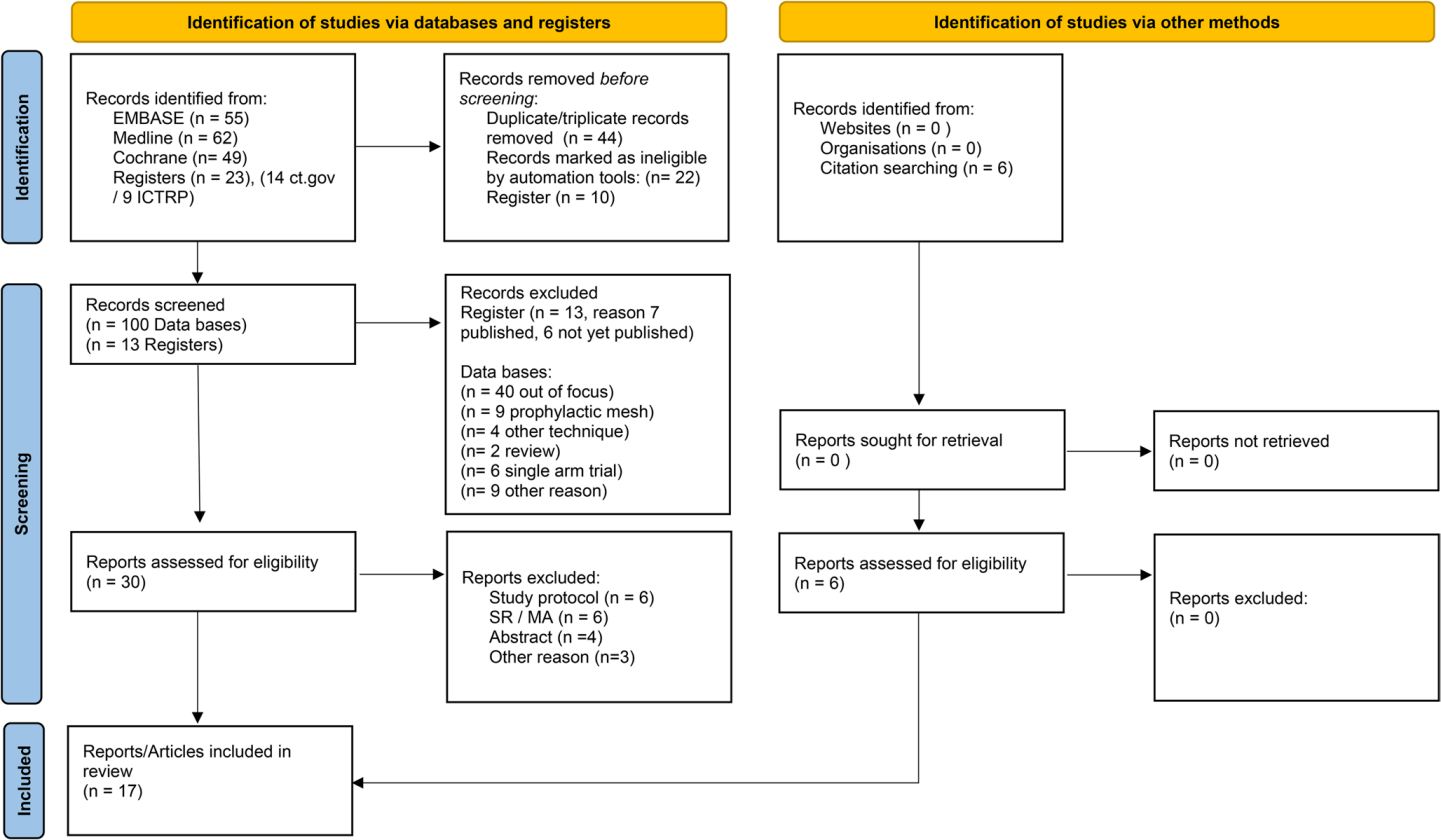
PRISMA Flow-Chart of SUBITE with regard to eligibility, screening and selection. Legend: CT.gov: clinicaltrials.gov, ICTRP: International Clinical Trials Registration Platform, MA: Meta-analysis, n: number, PRISMA: Preferred Reporting Items for Systematic reviews and Meta-Analysis, SR: Systematic Review

In Fig. 1, the PRISMA selection process is shown. Our initial search identified 189 results. After the removal of duplicates, registries and ineligible records marked by an automation tool, 113 records remained. The titles and abstracts of these records (*n* = 113) were screened based on inclusion criteria and a further 83 were excluded. Finally, 30 articles were assessed for eligibility and full-text review was performed leading to 11 articles, which met the inclusion criteria and were further systematically analyzed. The reference list of these 11 articles was checked and six additional publications found, resulting in a total of 17 articles including 10 RCTs and 6 comparative n-RCTs, (the 2 articles of ESTOIH study published by Albertsmeier et al. and Fortelny et al. were counted as 1 RCT) [7–9, 26–39].

### Methodological and quality assessment

**Fig. 2:**
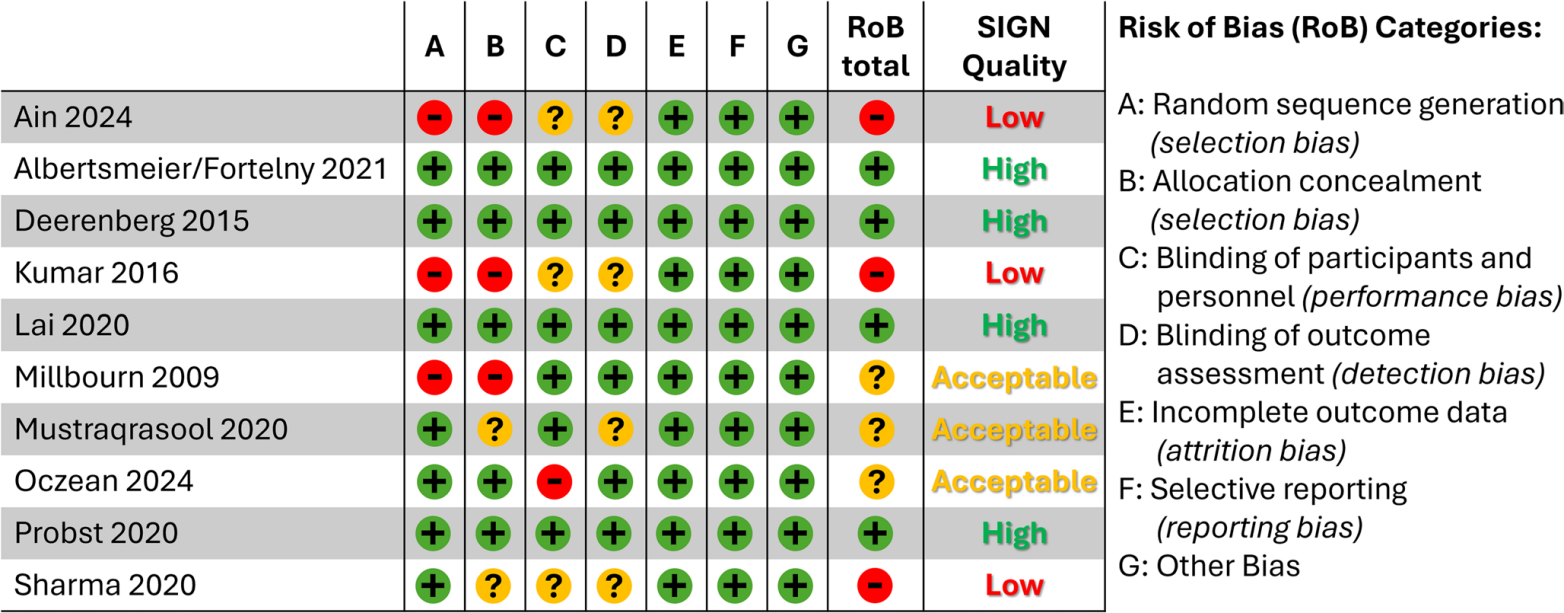
Assessment of RCTs according to RoB. Legend: RCT: Randomised Controlled Trials, RoB: Risk of Bias, (green = low risk, yellow = unclear risk, red = high risk)

The RoB evaluation of 10 RCTs is shown in Fig. 2. Using the RoB tool, in total four RCTs were judged with a low risk of bias [8, 9, 26–28], in three RCTs a high risk of bias was found due to inadequate randomization sequence generation and allocation concealment [26, 32, 33] and in another RCT the domain of `blinding of participants and researchers` was rated with high risk of bias because of an open label design with the lack of patient blinding [29]. Furthermore, the study by Mustaqrasool et al. lacked information regarding the domain `allocation concealment` & `blinding of outcome assessment` and the RCT by Sharma et al. gave no details with regard to the domains `allocation concealment`, `blinding of participants & researchers` & `blinding of outcome assessment` [30, 31].

**Fig. 3:**
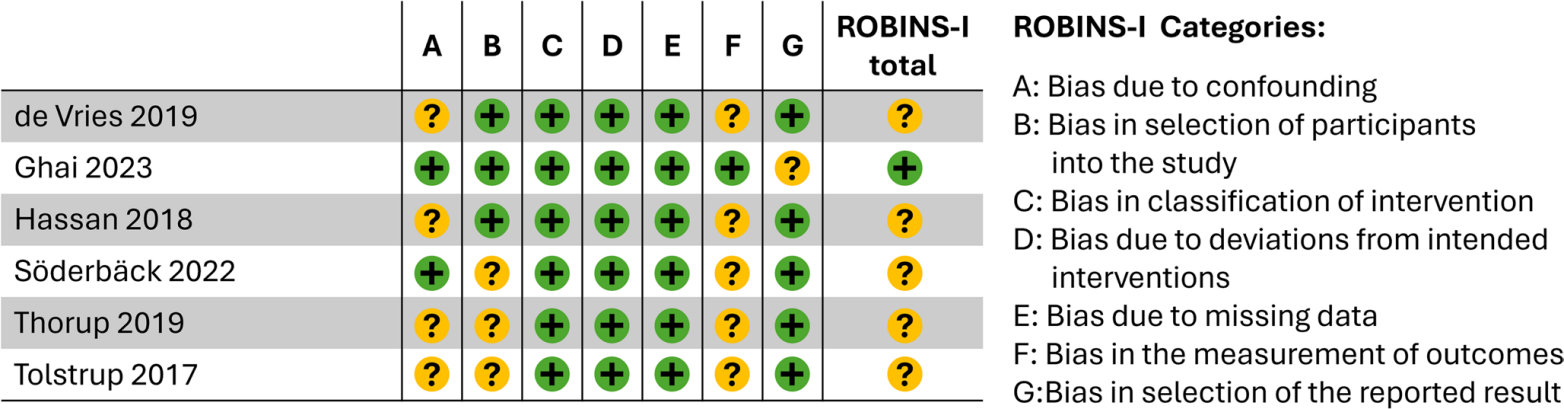
Assessment of n-RCTs according to ROBINS-1. Legend: n- RCT: non-Randomised Controlled Trials, Risk of Bias (green = low risk, yellow = unclear risk, red = high risk)

The MINORS scoring outcome of comparative n-RCTs is presented in Table 3. For our SR/MA we predefined a high (24 − 18 points), acceptable (17 − 12 points), low (11 − 6 points) & inacceptable quality score (5 − 0 points). Using this categorization, one low [36], two acceptable [37, 39] and three high quality [34, 35, 38] cohort studies (n-RCTs) were found. The ROBINS-I assessment indicated an overall moderate risk of bias (Fig. 3).

**Table 3:**
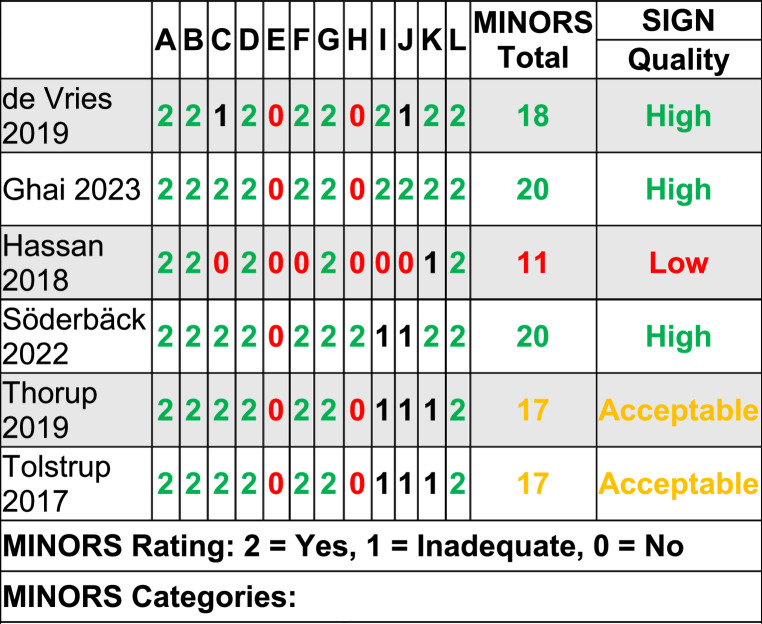
MINORS assessment of non-RCTs

A: A clearly stated aim

B: Inclusion of consecutive patients

C: Prospective collection of data

D: Endpoints appropriate to the aim of the study

E: Unbiased assessment of endpoint

F: Follow-up period appropriate to the aim of the study

G: Loss to follow up lessthan 5%

H: Prospective calculation of the study size

I: An adequate control group

J: Contemporary groups

K: Baseline equivalence of groups

L: Adequate statistical analysis

Legend:0= not reported, 1= inadequate reported, 2= adequate reported

According to the SIGN critical appraisal checklist, we classified five RCTs and three n-RCT as `high quality`, three RCTs and two n-RCTs as `acceptable quality` & three RCTs and one n-RCT as `low quality` studies (Table 2).

### Study characteristics

Study characteristics of all 16 studies (10 RCTs and 6 comparative non-RCTs) are summarized in Table 2. Year of publication extended from 2009 to 2024. Studies included clinics/departments in Europe (Germany, Austria, Sweden Denmark, Turkey) & Asia (India, Malaysia, Pakistan). The majority of studies had a monocentric design (*n* = 10) while six were multicentric. Total sample size varied from 60 to 1573 patients. Elective & emergency laparotomies as well as different types of suture materials (absorbable/ non-absorbable) were included. The small bite technique was described by a stitch interval of 5 mm and a lateral distance from the wound edge of 5–8 mm. For the large bite technique an inter-stitch interval of 10 mm and a lateral distance to the wound edge of 10 mm was utilized for mass closure or a linea alba only closure. Regarding comparative n-RCTs, the large/long bite group consisted of a historical control group, in which the applied suture technique was up to the surgeon´s preference (in 3 trials, i.e. before the adaption of the short stitch technique was introduced in these clinics/departments). The follow-up after surgery varied between 90 days and 52 months.

### Result of synthesis

Only studies that had a high or acceptable quality according to SIGN were included in our SR/MA. This requirement led to the final inclusion of 12 studies while four studies (three RCTs and one n-RCT, [Sharma et, Kumar et al., Ain et al., Hassan et al.]) had to be excluded. This sums up a population of 5886 patients recruited in 7 RCTs & 5 n-RCTs. The large bite group consisted of 3339, the short bite group of 2547 patients (Table 4).

**Table 4:**
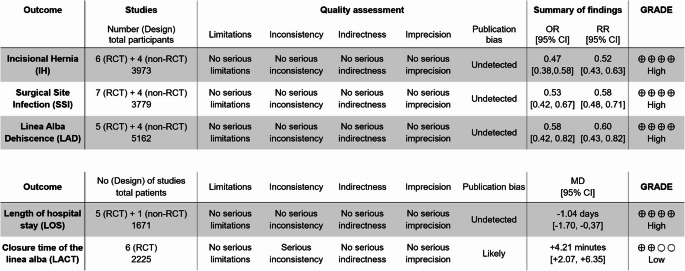
Assessment of all outcomes according to GRADE

Legend:*CI* Confidence interval, *GRADE* Grading of Recommendation Assessment,Development and Evaluation, *no* numbers, *n-RCT* non-Randomised Controlled Trials,*OR* Odds Ratio, *RCT* Randomised Controlled Trial, *RR* Relative Risk ratio

#### Incisional hernia (IH)

**Fig. 4:**
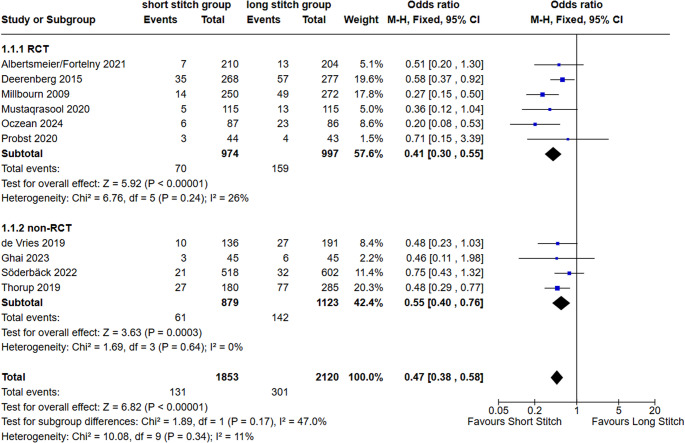
Forest plot of the primary outcome incisional hernia comparing short stitches vs. long stitches

All but one of the RCTs (*n* = 6) and four out of five comparative n-RCT reported incisional hernia rates (Fig. 4). According to these 10 studies incorporating 1853 patients in the short bite group (SB) and 2120 patients in the large bite group (LB), in total 131 cases of IH were observed in SB-, while 301 occurred in the LB- group. Thus, the IH rate was significantly lower in the SB- vs. LB-group in RCTs as well as n-RCTs (7.19% vs. 15.95% and 6.94% vs. 12.64% respectively). The overall odds ratio (OR) of incisional hernia was 0.47 (95% CI 0.38–0.58; *p* < 0.00001; I^2^ = 11%; Fig. 4). Subgroup differences between RCT and n-RCT studies were not significant (*p* = 0.17).

#### Surgical Site Infection (SSI)

**Fig. 5:**
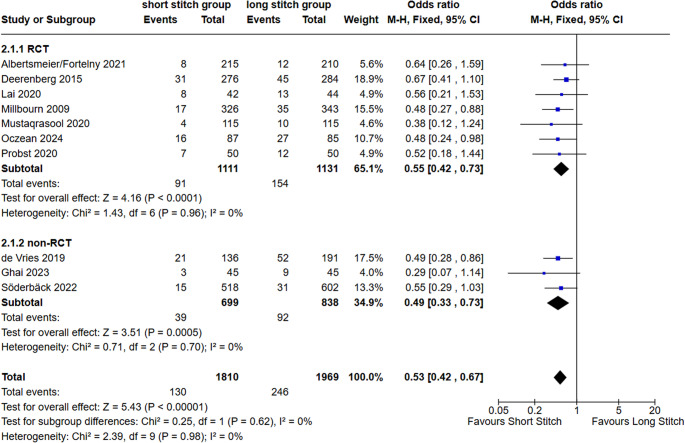
Forest plot of secondary outcome surgical site infection comparing short stitches vs. long stitches

Seven RCTs and three n-RCTs included SSI type A1 & A2 rates. Among these studies, in total 130 cases of SSIs were seen in the SB-, 246 cases in the LB-group. The overall odds ratio (OR) of SSIs was 0.53 (95% CI 0.42–0.67; *p* < 0.00001; I^2^ = 0%), indicating a significant benefit of the SB-technique for SSIs, independent of the study design (RCT or n-RCTs, *p* = 0.62; Fig. 5).

#### Linea alba dehiscence (LAD)

**Fig. 6:**
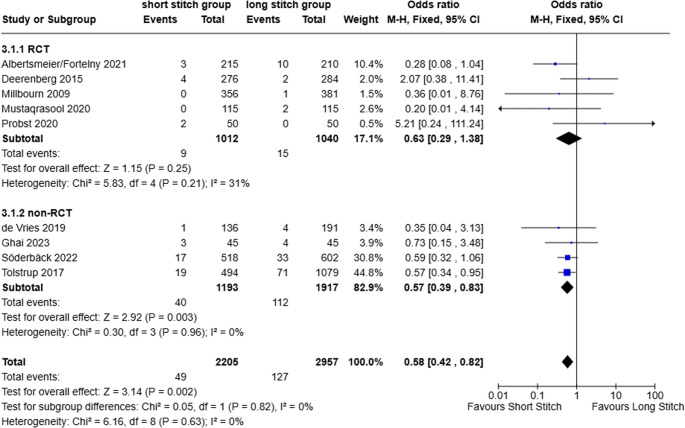
Forest plot of the secondary outcome linea alba dehiscence comparing short stitches vs. long stitches

Nine studies provided information about the occurrence of a linea alba dehiscence (RCT: *n* = 5, and n-RCT: *n* = 4). In total 49 linea alba dehiscence’s developed in the SB-, 127 in the LB-group. The total result of all trials (RCTs plus n-RCT) was significant, (*p* = 0.002), whereas the outcome of RCTs alone was insignificant (*p* = 0.25) and comparative n-RCT barely reached significance (*p* = 0.003). Again, with respect to linea alba dehiscence the SB-technique compared favorably to the LB-technique. The overall odds ratio (OR) of FD was 0.58 (95% CI 0.42–0.82; *p* = 0.002; I^2^ = 0%; Fig. 6) reducing the likelihood of a reoperation in the early phase by 42%.

#### Length of Hospital stay (LOS)

**Fig. 7:**
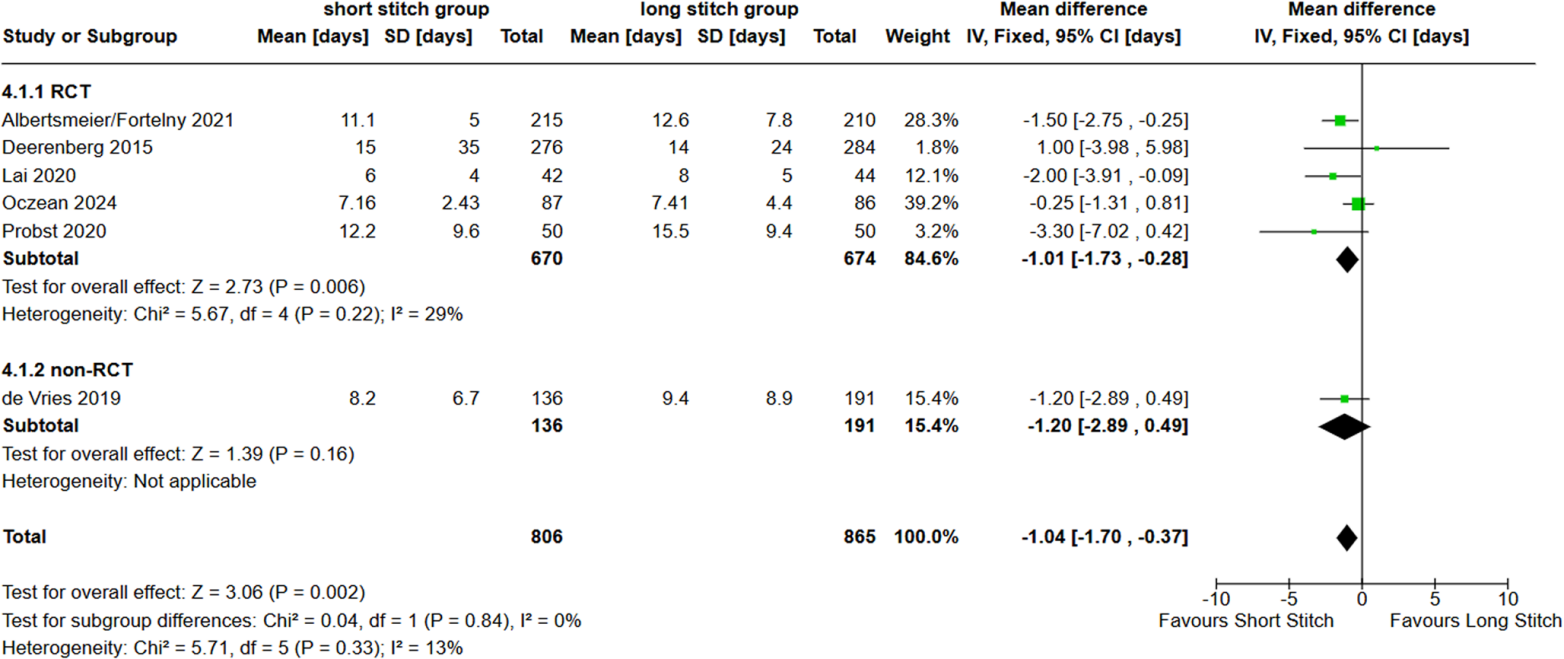
Forest plot of the secondary outcome length of hospital stay comparing short stitches vs. long stitches

Six studies (5 RCTs and 1 n-RCT) assessed the length of hospital stay. The length of hospital stay was significantly shorter in the SB- than LB-group (MD -1.04 (95% CI -1.70, -0.37; *p* = 0.002; I^2^ = 13%; Fig. 7).

#### Closure time of the linea alba

**Fig. 8:**
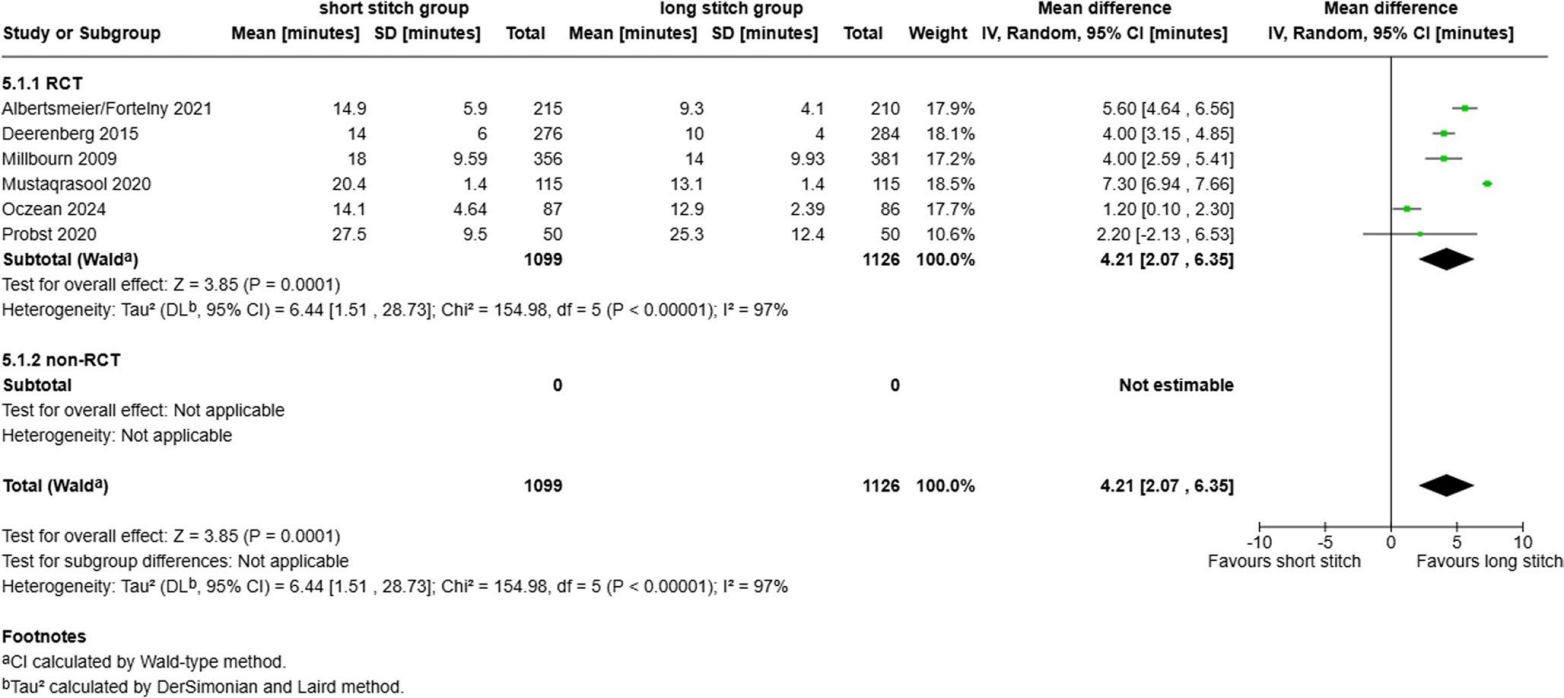
Forest plot of the secondary outcome time to close the linea alba comparing short stitch vs. long stitch

In total, six RCTs published data on the duration of fascial closure. SB-technique was associated with a significantly longer closure time (about 4.2 min. longer) compared to the LB-technique (MD 4.21 (95% CI 2.07–6.35; *p* = 0.0001; I^2^ = 97%; Fig. 8). Contrary to the other analysis, these results showed high heterogeneity in between studies (*p* < 0.00001).

#### Subgroup analysis

The subgroup analysis of the main outcome parameters (IH, SSI, & FD) depending on study design (mono- vs. multicentric), intervention type (primary vs. relaparotomy, elective vs. emergency), and closure method (mass or linea alba only closure) indicated in all cases the superiority of the SB- over the LB-technique (data not shown). Furthermore, explicit results on the impact of the suture material (absorption profile, elasticity, tensile strength) will be published in the near future.

### Reporting bias and certainty of evidence

**Fig. 9:**
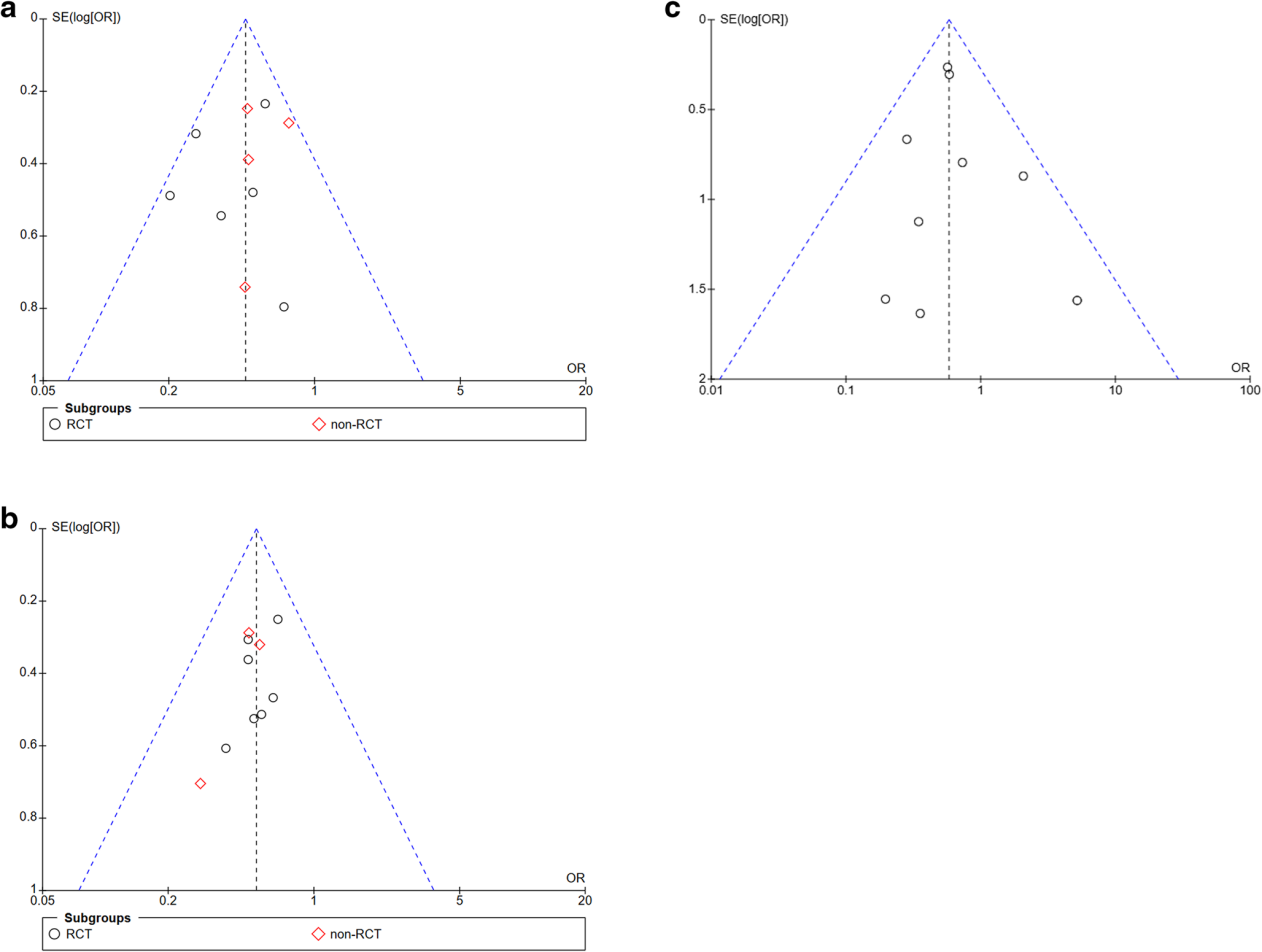

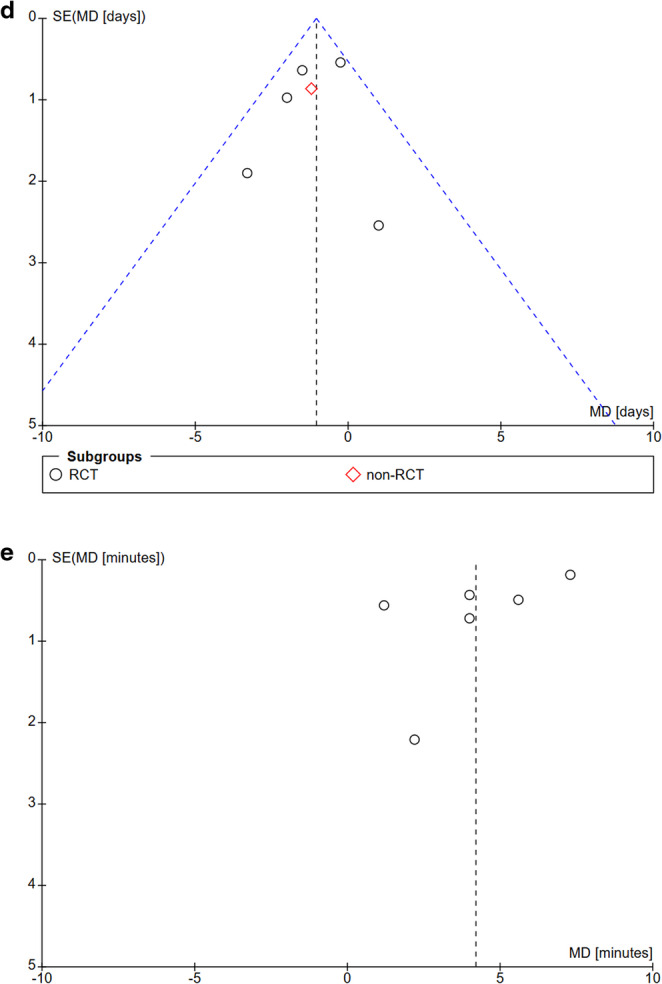
Publication bias funnel plot. Legend: Funnel Plots (**a**) incisional hernia, (**b**) surgical site infection, (**c**) linea alba dehiscence, (**d**) length of hospital stay, (**e**) time to close the linea alba

To assess a possible publication or reporting bias (> 10 studies) we chose the graphic illustration with funnel plots. In Fig. 9 (a, b, c, d), a symmetrical distribution of RCTs & n-RCTs with regard to `IH, SSI, FD & LOS` illustrates no significant publication or reporting bias. Only the parameter `closure time` showed a heterogeneity of I^2^ = 97% in forest-plots implying a reporting bias (Fig. 9e).

The summary of the confidence of the resulting body of evidence using the established and validated GRADE-tool indicated a high level of evidence for the categories IH, SSI, LAD, and LOS (Table 4). Only for the category `duration of fascial closure ` showed a serious inconsistency and a likely publication bias leading to a low-quality GRADE rating.

## Discussion

### Strength and consistency of evidence

The available evidence suggests consistent findings in favor of the small bites technique for midline laparotomy closure. Several independent meta-analyses, primarily based on randomized controlled trials, have reported similar effect directions, indicating a reduction in incisional hernia and surgical site infection compared with the large bites technique [11, 14–17]. Our systematic review and meta-analysis (SUBITE) add to this body of evidence by integrating data from both randomized and non-randomized comparative studies. The inclusion of seven RCTs and five non-randomized studies generating `real world data` may enhance external validity; however, the inherent limitations of observational designs, including potential confounding, must be acknowledged. Although publication bias was detected in some non-randomized studies, pooled effect estimates remained broadly consistent with those derived from randomized trials. Additional assurance is conferred by the use of a trial sequential analysis by Alrashidi et al. (2025) which adds evidence to draw the definitive conclusion that further trials are unlikely to change the overall direction of the findings [15].

General quality of the underlying primary studies is high, with landmark trials such as STITCH and ESTOIH employing rigorous double-blind, multicentre randomized controlled trial designs [8, 9].

### Long-term outcomes and durability

One of the most important contributions of recent research has been the publication of long-term follow-up data from the ESTOIH trial. The 3-year and 5-year follow-up analyses by Fortelny et al. (2024, 2025) confirms that early benefits can be sustained over time [46, 47]. This long-term evidence is particularly important because the incidence of incisional hernia continues to increase for several years after surgery resulting in cumulative incidence rates as high as 60% by 3 to 5 years [16, 48]. Thus, persistent benefits would counter the argument that the small bite merely postpones hernia occurrence rather than providing genuine prevention [46].

### Mechanisms of benefit

The mechanisms underlying superior outcomes with the small bites/short stitch technique are likely multifactorial. From a biomechanical perspective, incorporating only the strong aponeurotic layer in small, closely spaced bites distributes tension more evenly along the suture line and avoids the weaker muscle tissue [48]. From a biological perspective, the small bites technique minimizes tissue devitalization and ischemia by taking smaller tissue bites and avoiding muscle incorporation. Reduced tissue trauma may promote better wound healing and reduce the inflammatory response that can compromise linea alba integrity [17]. Giving the known association between SSI and subsequent hernia formation, the technique likely provides both direct mechanical and indirect benefits through improved wound healing [37].

### Implementation barriers and practice variation

Despite the strong and consistent evidence favoring the small bites technique, adoption in clinical practice has been disappointing. Lawday et al. (2024) conducted a qualitative study examining whether randomized evidence alters clinical practice, specifically in the context of the STITCH trial [49]. Qualitative research has identified several barriers to implementation, including (a) lack of awareness of the evidence among some surgeons, (b) resistance to changing established practice patterns, (c) concerns about the time required to perform the small bites/short stitch technique, (d) insufficient emphasis on closure technique in surgical training and (e) organizational and cultural factors in surgical departments.

It can be noted, that despite the small bites technique being recognized as the standard, the large bites technique remains common in practice [16]. This evidence-practice gap represents a significant opportunity for quality improvement and patient safety initiatives. The implementation study by Vries et al. (2020) demonstrated that when the small bites technique is successfully implemented in clinical practice, the expected benefits in terms of reduced surgical site infections and incisional hernias are realized [34]. This real-world evidence suggests that the trial findings are generalizable and achievable in routine practice when appropriate implementation strategies are employed.

### Special populations and clinical contexts

Two trials addressed the use of small bites in contaminated cases, overweight patients and gynecological malignancies [50, 51] suggesting benefits can be generalized across different patient populations and surgical contexts. The question of whether prophylactic mesh augmentation should be combined with small bites closure in high-risk patients remains an area of active investigation. The factorial design employed by Coelho et al. (2023) provides a methodological framework for evaluating such combinations [51].

### Patient-centered outcomes

The pooled analysis demonstrated consistent effect estimates across multiple clinically relevant outcomes, including incisional hernia, surgical site infection, and linea alba dehiscence. Statistical heterogeneity in the analysis of IH, SSI, FD and LOS were low. Only closure time showed a relevant publication bias and a high heterogeneity.

While the focus of most studies has been on objective outcomes (IH, SSI), patient-centered outcomes are also important. The study by Lai et al. (2021) specifically addressed the concern that the more frequent suture passes in the short stitch might increase pain [27]. The prevention of IH has significant implications for quality of life, including reduced chronic pain, functional limitations, body image, and the need for additional surgery [8].

### Economic considerations

While formal cost-effectiveness analyses were not included in the reviewed studies, the economic implications of the small bite technique are likely favorable. Preventing hernias through improved closure technique is a cost-effective intervention, does not require specialized equipment or materials, making it accessible & affordable [49]. The reduction in SSI shown strengthens the economic case for adoption, since SSI is associated with prolonged hospitalization, additional treatments, and increased healthcare costs [37].

### Limitations and areas of uncertainty

Despite the strong evidence base, several limitations and areas of uncertainty remain.

*First*, the meta-analytic evidence in SUBITE for reduced *linea alba dehiscence* with the small bites technique is suggestive (OR 0.58). Nevertheless, the confidence interval related to the secondary wound dehiscence and burst abdomen in some RCTs crosses unity [7–9, 28, 30] and due to lower numbers may not be statistically significant. The relatively higher incidence of FD corporating both target parameters may provide statistical power to detect potential significance. *Second*, while long-term follow-up data from the ESTOIH trial extend to 5 years, even longer follow-up may be valuable given that some hernias develop many years after surgery. Continued follow-up of trial cohorts would provide additional information on even longer outcomes [47]. *Third*, subgroup analyses examining the effectiveness of the small bites technique in specific high-risk populations (e.g., obese patients, emergency surgery, contaminated fields) are limited [51, 52]. *Fourth*, it is important to mention that this technique was exclusively studied in median incisions. This has technical and anatomical implications. Whether the results can be applied to transverse incisions is at least questionable [52]. In addition, an increase of the SL/WL-ratio (> 5:1) may assure that an > 4:1 SL/WL can definitely be achieved and might further improve results. *Finally*, the optimal suture material to use with the small bites technique remains an area of investigation. Some trials used an ultra-long-term absorbable elastic monofilament suture, but whether this specific material is superior to other options needs to be clarified [9, 52].

### Conclusion & outlook: evidence guidelines and implications for surgical training and education

Current evidence including SUBITE systematic review and meta-analysis supports strong consideration of the small bites technique for midline laparotomy closure within guideline frameworks and surgical training programs. Given that this technique should now be considered the standard of care for midline laparotomy closure, it should be emphasized in surgical residency training programs and continuing medical education for practicing surgeons [48]. Simulation-based training and video demonstrations may be valuable tools for teaching the technical aspects of the small bites technique. Ensuring that trainees develop proficiency with this technique early in their training will help ensure widespread adoption in future surgical practice [49]. Quality improvement initiatives at the institutional level, including audit and feedback on closure technique and outcomes, may help drive adoption of evidence-based practices. The implementation study by de Vries et al. (2020) provides a role model for how systematic implementation of the small bites/short stitch technique can be achieved in clinical practice [34]. Future research should focus on long-term durability, high-risk populations, cost-effectiveness, and optimization of technical parameters to further refine its role in contemporary surgical practice. Future research should focus on long-term durability, high-risk populations, cost-effectiveness, and optimization of technical parameters to further refine its role in contemporary surgical practice.

Importantly, while previous meta-analyses have consistently demonstrated advantages of the small bites technique, their impact on clinical practice has been limited, likely due to restricted study inclusion, heterogeneity, and limited external validity. By integrating both randomized and well-designed comparative real-world data, the present analysis addresses these limitations and provides a more robust and practice-oriented evidence base, which may facilitate broader implementation of the technique in routine surgical practice.

A second part of the SUBITE meta-analysis will further assess whether suture material characteristics—particularly absorption profile, elasticity, and tensile strength—affect clinical outcomes when combined with the short bites technique after midline laparotomy closure.

## Supplementary Information

Below is the link to the electronic supplementary material.

Supplementary Material 1

Supplementary Material 2

Supplementary Material 3
