# Supplementary material for: Impact of the SUture BIte TEchnique on clinical outcomes after midline laparotomy closure: SUBITE—a systematic review and meta-analysis
Source: Hernia. 2026 May 19;30(1):221. doi: 10.1007/s10029-026-03700-z (PMC13186860; doi:10.1007/s10029-026-03700-z)
Supplement: Supplementary file 1 — Supplementary Material 1 [file 10029_2026_3700_MOESM1_ESM.docx]

S1: Embase

### Embase session results (10 Jan 2025), 18:56 MEZ

| No. | Query | Results |
| --- | --- | --- |
| #1 | 'laparotomy'/exp OR 'abdomen laparotomy' OR 'abdominal laparotomy' OR 'celiotomy' OR 'coeliotomy' OR 'laparotomy' OR 'midline incision'/exp OR '`abdominal wall incision`' OR 'abdominal wound' | 136033 |
| #2 | 'suture'/exp OR 'open-surgery ligature loop' OR 'surgical suture' OR 'surgical suture, device' OR 'surgical suture, device (physical object)' OR 'surgical suture, nos' OR 'surgical sutures' OR 'surgical sutures (physical object)' OR 'suture' OR 'suture (physical object)' OR 'suture - object' OR 'sutures' OR 'wound closure'/exp OR 'closure, wound' OR 'wound closure' OR 'wound closure techniques' OR 'suture technique'/exp OR 'suture technique' OR 'suture techniques' OR 'suturing method' OR 'fascial closure'/exp OR 'abdominal closure'/exp OR '`midline closure`' | 218035 |
| #3 | ('small' OR 'short' OR 'big' OR 'large' OR 'long') AND ('stitch' OR 'stitch*' OR 'bit' OR 'bites' OR step*) | 466641 |
| #4 | 'postoperative complication'/exp OR 'complication after operation' OR 'complication after surgery' OR 'complication, postoperative' OR 'complication, surgical' OR 'post-operation complication' OR 'post-operative complication' OR 'post-operative complications' OR 'post-surgery complication' OR 'post-surgical complication' OR 'postoperation complication' OR 'postoperative complication' OR 'postoperative complications' OR 'postsurgery complication' OR 'postsurgical complication' OR 'surgery complication' OR 'surgery complications' OR 'surgery-associated complication' OR 'surgery-derived complication' OR 'surgery-induced complication' OR 'surgery-related complication' OR 'surgical complication' OR 'hernia'/exp OR 'hernia' OR 'herniation' OR 'burst abdomen' OR 'wound dehiscence'/exp OR 'burst wound' OR 'dehisced incision' OR 'dehisced scar' OR 'dehisced surgical incision' OR 'dehisced surgical wound' OR 'dehisced suture' OR 'dehisced wound' OR 'dehiscence, wound' OR 'dehiscent incision' OR 'dehiscent scar' OR 'dehiscent surgical wound' OR 'dehiscent suture' OR 'dehiscent wound' OR 'disrupted operation wound' OR 'disrupted operative wound' OR 'disrupted surgical incision' OR 'disrupted surgical wound' OR 'disrupted wound' OR 'ruptured incision' OR 'ruptured operation wound' OR 'surgical wound dehiscence' OR 'suture dehiscence' OR 'wound dehiscence' OR 'wound disruption' OR 'wound rupture' OR 'wound separation' OR 'surgical infection'/exp OR 'ssi (surgical site infection)' OR 'infected surgical site' OR 'infected surgical wound' OR 'infection at the operative site' OR 'infection at the surgery site' OR 'infection at the surgical site' OR 'infection of the operative site' OR 'infection of the surgery site' OR 'infection of the surgical site' OR 'infection of the surgical wound' OR 'infection, surgical' OR 'operative site infection' OR 'post-operative wound infection' OR 'post-operative wound infections' OR 'postoperative wound infection' OR 'postoperative wound infections' OR 'surgery site infection' OR 'surgical infection' OR 'surgical infections' OR 'surgical site infection' OR 'surgical wound infection' OR 'surgical wound infections' OR 'ssi' OR (('incisional' OR 'ventral' OR 'wound') AND 'complication*') | 1327166 |
| #5 | #1 AND #2 AND #3 AND #4 | 222 |
| #6 | [randomized controlled trial]/lim OR [controlled clinical trial]/lim OR randomized:ab,ti OR 'clinical trials as topic'/de OR randomly:ab,ti OR random:ab,ti OR random*:ab,ti OR trial:ab,ti | 3072388 |
| #7 | #5 AND #6 | 63 |
| #8 | [animals]/lim NOT [humans]/lim | 6626438 |
| #9 | #7 NOT #8 | 55 |

## S2: MEDLINE

Search: January 10, 2025, 19:03 MEZ

| **No** | Query | Results |
| --- | --- | --- |
| **#1** | „laparotomy“[Mesh Terms] OR „abdomen laparotomy“ OR „abdominal laparotomy“ OR „celiotomy“ OR „coeliotomy“ OR „laparotomy“ OR „midline incision“ OR „abdominal wall incision“ OR „abdominal wound” | 69.622 |
| **#2** | „sutures“[Mesh Terms] OR „surgical suture“ OR „surgical sutures“ OR „suture“ OR „sutures“ OR „abdominal wound closure techniques“[Mesh Terms] OR „closure, wound“ OR „wound closure“ OR „wound closure techniques“ OR „suture techniques“[MeSH Terms] OR „suture technique“ OR „suture techniques“ OR „suturing method“ OR „fascial closure“ OR „abdominal closure“ OR „midline closure” | 127.551 |
| #3 | („small“ OR „short“ OR „big“ OR „large“ OR „long“) AND („stitch“ OR stitch* OR „bit“ OR „bites“ OR „step“ OR step*)" | 238.956 |
| #4 | („postoperative complications“[MeSH Terms] OR „complication after operation“ OR „complication after surgery“ OR „complication, postoperative“ OR „complication, surgical“ OR „post-operation complication“ OR „post-operative complication“ OR „post-operative complications“ OR „post-surgery complication“ OR „post-surgical complication“ OR „postoperation complication“ OR „postoperative complication“ OR „postoperative complications“ OR „postsurgery complication“ OR „postsurgical complication“ OR „surgery complication“ OR „surgery complications“ OR „surgery-associated complication“ OR „surgery-derived complication“ OR „surgery-induced complication“ OR „surgery-related complication“ OR „surgical complication“ OR „hernia“[MeSH Terms] OR „hernia“ OR „herniation“ OR „burst abdomen“ OR „wound healing/complications“[MeSH Terms] OR „burst wound“ OR „dehisced incision“ OR „dehisced scar“ OR „dehisced surgical incision“ OR „dehisced surgical wound“ OR „dehisced suture“ OR „dehisced wound“ OR „dehiscence, wound“ OR „dehiscent incision“ OR „dehiscent scar“ OR „dehiscent surgical wound“ OR „dehiscent suture“ OR „dehiscent wound“ OR „disrupted operation wound“ OR „disrupted operative wound“ OR „disrupted surgical incision“ OR „disrupted surgical wound“ OR „disrupted wound“ OR „ruptured incision“ OR „ruptured operation wound“ OR „surgical wound dehiscence“ OR „suture dehiscence“ OR „wound dehiscence“ OR „wound disruption“ OR „wound rupture“ OR „wound separation“ OR „infected surgical site“ OR „infected surgical wound“ OR „infection at the operative site“ OR „infection at the surgery site“ OR „infection at the surgical site“ OR „infection of the operative site“ OR „infection of the surgery site“ OR „infection of the surgical site“ OR „infection of the surgical wound“ OR „infection, surgical“ OR „operative site infection“ OR „post-operative wound infection“ OR „post-operative wound infections“ OR „postoperative wound infection“ OR „postoperative wound infections“ OR „surgery site infection“ OR „surgical infection“ OR „surgical infections“ OR „surgical site infection“ OR „surgical wound infection“ OR „surgical wound infections“ OR „ssi“ OR ((„incisional“ OR „ventral“ OR „wound“) AND complication | 1.343.182 |
| #5 | #1 AND #2 AND #3 AND #4 | 143 |
| #6 | "Randomized Controlled Trial“[Publication Type] OR „controlled clinical trial“ [Publication Type] OR randomized[TIAB] OR „Clinical Trials as Topic“[Mesh:NoExp] OR randomly[TIAB] OR random[TIAB] OR random*[TIAB] OR trial[TIAB] OR 'clinical study' OR 'randomized controlled study' OR 'randomised controlled study' OR 'comparative study' OR 'cohort study' | 6.582.404 |
| #7 | #5 AND #6 | 71 |
| #8 | #5 AND #6, Filter: Humans | 62 |

## S3: Cochrane

| Search Name: |  |  |
| --- | --- | --- |
| Date Run: | 13/01/2025 15:34:14 |  |
| Comment: |  |  |
|  |  |  |
| ID | Search | Hits |
| #1 | MeSH descriptor: [Laparotomy] explode all trees | 1033 |
| #2 | MeSH descriptor: [Abdominal Injuries] explode all trees | 205 |
| #3 | „midline incision" OR „abdominal wall incision" | 908 |
| #4 | #1 OR #2 OR #3 | 2053 |
| #5 | MeSH descriptor: [Suture Techniques] explode all trees | 2771 |
| #6 | „wound closure techniques“ OR „suture technique“ OR „suture techniques“ OR „suturing method“ | 4506 |
| #7 | „abdominal closure“ OR „midline closure” OR "fascial closure" | 2169 |
| #8 | #5 OR #6 OR #7 | 6453 |
| #9 | ((long OR big OR large OR short OR small) AND (stitch* OR step* OR bit*)) | 20328 |
| #10 | #8 AND #9 | 412 |
| #11 | MeSH descriptor: [Postoperative Complications] explode all trees | 56840 |
| #12 | MeSH descriptor: [Hernia] explode all trees | 4544 |
| #13 | MeSH descriptor: [Wound Healing] explode all trees | 8428 |
| #14 | "complication" OR "hernia" OR "Wound Dehiscence" OR "Burst Abdomen" OR "Surgical Site Infection" OR "SSI" | 104541 |
| #15 | #11 OR #12 OR #13 OR #14 | 152952 |
| #16 | #4 AND #10 AND #15 | 72 |
|  |  |  |

21 Cochrane Reviews

1 Cochrane Protocol

50 Clinical Trials in CENTRAL (Cochrane Central Register of Controlled Trials)

27 Journal Articles

23 Registry Records (14 ct.gov, 9 ICTRP)

1

### [Comparison of Post-operative Pain in Short Versus Long Stitch for Laparotomy Closure](https://www.cochranelibrary.com/central/doi/10.1002/central/CN-01796224/full)

NCT03828409

https://clinicaltrials.gov/show/NCT03828409, **2019** | added to CENTRAL: 31 March 2019 | 2019 Issue 3

CT.gov

2

### [Small Bite Technique Versus Standardised Large Bites Technique in Closure of Midline Laparotomies](https://www.cochranelibrary.com/central/doi/10.1002/central/CN-02249728/full)

NCT04788875

https://clinicaltrials.gov/show/NCT04788875, **2021** | added to CENTRAL: 31 March 2021 | 2021 Issue 3

CT.gov

3

### [Reducing the Incidence of Incisional Hernia After Stoma Closure Using a Prophylactic Mesh](https://www.cochranelibrary.com/central/doi/10.1002/central/CN-02146068/full)

NCT04510558

https://clinicaltrials.gov/show/NCT04510558, **2020** | added to CENTRAL: 31 August 2020 | 2020 Issue 08

CT.gov

4

### [Suture Techniques to Reduce the Incidence of Incisional Hernia: LTFU STITCH Trial](https://www.cochranelibrary.com/central/doi/10.1002/central/CN-02602326/full)

NCT06066385

https://clinicaltrials.gov/ct2/show/NCT06066385, **2023** | added to CENTRAL: 31 October 2023 | 2023 Issue 10

CT.gov

5

### [Barbed Suture vs Non-Barbed Closure for Emergency Exploratory Laparotomy RCT](https://www.cochranelibrary.com/central/doi/10.1002/central/CN-02598588/full)

NCT06043414

https://clinicaltrials.gov/ct2/show/NCT06043414, **2023** | added to CENTRAL: 30 September 2023 | 2023 Issue 9

CT.gov

6

### [Comparing Wound Complications After Elective Abdominal Surgery Using Two Closure Techniques](https://www.cochranelibrary.com/central/doi/10.1002/central/CN-01659596/full)

NCT03527433

https://clinicaltrials.gov/ct2/show/NCT03527433, **2018** | added to CENTRAL: 31 January 2019 | 2019 Issue 1

CT.gov

7

### [Preemer Trial - Prophylactic Mesh Versus no Mesh in the Midline Emergency Laparotomy Closure for Prevention of Incisional Hernia: a Multi Center, Double-blind, Randomized Controlled Trial](https://www.cochranelibrary.com/central/doi/10.1002/central/CN-02089160/full)

NCT04311788

https://clinicaltrials.gov/ct2/show/NCT04311788, **2020** | added to CENTRAL: 31 March 2020 | 2020 Issue 03

CT.gov

8

### [A Non-Traumatic Binder for Temporary Abdominal Wall Closure](https://www.cochranelibrary.com/central/doi/10.1002/central/CN-01796040/full)

NCT03815370

https://clinicaltrials.gov/show/NCT03815370, **2017** | added to CENTRAL: 31 March 2019 | 2019 Issue 3

CT.gov

9

### [DuraMesh Laparotomy Study](https://www.cochranelibrary.com/central/doi/10.1002/central/CN-01945136/full)

NCT03966768

https://clinicaltrials.gov/show/NCT03966768, **2019** | added to CENTRAL: 30 June 2019 | 2019 Issue 06

CT.gov

10

### [When Closing Midline Incisions, do Small Stitches Reduce the Risk for Incisional Hernia, Wound Infection or Dehiscence?](https://www.cochranelibrary.com/central/doi/10.1002/central/CN-02013811/full)

NCT00508053

https://clinicaltrials.gov/show/NCT00508053, **2007** | added to CENTRAL: 31 January 2020 | 2020 Issue 01

CT.gov

11

### [Hernia After Colorectal Cancer Surgery](https://www.cochranelibrary.com/central/doi/10.1002/central/CN-01567204/full)

NCT03390764

https://clinicaltrials.gov/show/NCT03390764, **2017** | added to CENTRAL: 31 May 2018 | 2018 Issue 5

CT.gov

12

### [Laparoscopic Inguinal Hernia Repair- Transabdominal Preperitoneal (TAPP) Versus Totally Extra Peritoneal (TEP)](https://www.cochranelibrary.com/central/doi/10.1002/central/CN-01519056/full)

NCT00687375

https://clinicaltrials.gov/show/NCT00687375, **2008** | added to CENTRAL: 31 May 2018 | 2018 Issue 5

CT.gov

13

### [Examining the Cosmetic Results, Quality of Life and Patient Satisfaction Achieved With Skin Reducing Nipple Sparing Mastectomy and Implant Based Breast Reconstruction, and Comparing it to Classic Skin- Sparing Mastectomy and Implant- Based Breast Reconstructive Surgeries](https://www.cochranelibrary.com/central/doi/10.1002/central/CN-02180392/full)

NCT04345081

https://clinicaltrials.gov/show/NCT04345081, **2020** | added to CENTRAL: 31 October 2020 | 2020 Issue 10

CT.gov

14

### [MIRRAD Versus Plication of Entire Diastasis Trial](https://www.cochranelibrary.com/central/doi/10.1002/central/CN-02531316/full)

NCT05759663

https://clinicaltrials.gov/show/NCT05759663, **2023** | added to CENTRAL: 31 March 2023 | 2023 Issue 3

CT.gov

1

### [Suture Techniques to reduce the Incidence of The inCisional Hernia](https://www.cochranelibrary.com/central/doi/10.1002/central/CN-02714524/full)

NL-OMON37004

https://trialsearch.who.int/Trial2.aspx?TrialID=NL-OMON37004, **2009** | added to CENTRAL: 30 June 2024 | 2024 Issue 6

ICTRP

2

### [Abdominal closure after emergency laparotomy](https://www.cochranelibrary.com/central/doi/10.1002/central/CN-02684515/full)

CTRI/2024/03/064359

https://trialsearch.who.int/Trial2.aspx?TrialID=CTRI/2024/03/064359, **2024** | added to CENTRAL: 30 April 2024 | 2024 Issue 4

ICTRP

3

### [Small bite technique versus large bite technique for surgical incision closure in preventing incisional hernia in gynaec cancer patients undergoing surgery by a midline vertical incision : a Randomised controlled trial](https://www.cochranelibrary.com/central/doi/10.1002/central/CN-02377488/full)

CTRI/2022/01/039700

https://trialsearch.who.int/Trial2.aspx?TrialID=CTRI/2022/01/039700, **2022** | added to CENTRAL: 31 March 2022 | 2022 Issue 03

ICTRP

4

### [Comparison of two different types of abdomen closure after surgery](https://www.cochranelibrary.com/central/doi/10.1002/central/CN-02591309/full)

CTRI/2023/07/055903

https://trialsearch.who.int/Trial2.aspx?TrialID=CTRI/2023/07/055903, **2023** | added to CENTRAL: 31 August 2023 | 2023 Issue 8

ICTRP

5

### [Comparison study of short stitch and conventional stitch technique for midline closure](https://www.cochranelibrary.com/central/doi/10.1002/central/CN-02674710/full)

CTRI/2024/02/062597

https://trialsearch.who.int/Trial2.aspx?TrialID=CTRI/2024/02/062597, **2024** | added to CENTRAL: 31 March 2024 | 2024 Issue 3

ICTRP

6

### [Small bite continuous suture closure vs. mass closure of midline abdominal incision in emergency(SMILE Trial)](https://www.cochranelibrary.com/central/doi/10.1002/central/CN-02168554/full)

CTRI/2020/06/026210

https://trialsearch.who.int/Trial2.aspx?TrialID=CTRI/2020/06/026210, **2020** | added to CENTRAL: 31 October 2020 | 2020 Issue 10

ICTRP

7

### [Hernia reduction following laparotomy using small stitch abdominal wall closure with or without mesh augmentation](https://www.cochranelibrary.com/central/doi/10.1002/central/CN-01973815/full)

DRKS00017517

https://trialsearch.who.int/Trial2.aspx?TrialID=DRKS00017517, **2019** | added to CENTRAL: 30 September 2019 | 2019 Issue 09

ICTRP

8

### [Midline incisional hernia prophylaxis using synthetic mesh in emergency or urgency gastrointestinal tract surgery: a multicenter randomized clinical trial](https://www.cochranelibrary.com/central/doi/10.1002/central/CN-02190075/full)

TCTR20200924002

https://trialsearch.who.int/Trial2.aspx?TrialID=TCTR20200924002, **2020** | added to CENTRAL: 30 November 2020 | 2020 Issue 11

ICTRP

9

### [Suture Techniques to reduce the Incidence of The inCisional Hernia](https://www.cochranelibrary.com/central/doi/10.1002/central/CN-01863978/full)

NTR2052

https://trialsearch.who.int/Trial2.aspx?TrialID=NTR2052, **2009** | added to CENTRAL: 31 March 2019 | 2019 Issue 3

ICTRP

Formularbeginn

Formularende
