# Supplementary material for: Impact of the SUture BIte TEchnique on clinical outcomes after midline laparotomy closure: SUBITE—a systematic review and meta-analysis
Source: Hernia. 2026 May 19;30(1):221. doi: 10.1007/s10029-026-03700-z (PMC13186860; doi:10.1007/s10029-026-03700-z)
Supplement: Supplementary file 3 — Supplementary Material 3 [file 10029_2026_3700_MOESM3_ESM.docx]

**Competing interest**

PB and FK are employed by Aesculap AG.

MG has received research funding by B.Braun, when involved in prospective studies/trials as well personal reimbursements for scientific presentations. This is irrespective of his research validity and the coauthor ship of this Systematic Review & Meta analysis. Moreover, he has received contributions for presentations by other medical companies such as Baxter, Becton Dickinson, Falk, Gore, Johnson & Johnson and Medtronic. He is not financially involved in any of these companies.

RF reports having received research funding from B. Braun and Becton Dickinson for involvement in clinical studies within the last 36 months. RF further reports having received honoraria for scientific presentations and educational activities from Fasciotens and Medtronic within the last 36 months. These relationships are unrelated to the content, design, analysis, interpretation, or reporting of the present manuscript. RF declares no consultancy roles, no advisory board memberships, no equity interests, no stock ownership, and no other financial or non-financial conflicts of interest.
