# Supplementary material for: Impact of the SUture BIte TEchnique on clinical outcomes after midline laparotomy closure: SUBITE—a systematic review and meta-analysis
Source: Hernia. 2026 May 19;30(1):221. doi: 10.1007/s10029-026-03700-z (PMC13186860; doi:10.1007/s10029-026-03700-z)
Supplement: Supplementary file 2 — Supplementary Material 2 [file 10029_2026_3700_MOESM2_ESM.docx]

Dear Editor and Reviewers,

We would like to thank the reviewers for their thorough evaluation of our manuscript and for the constructive and insightful comments. We have carefully revised the manuscript accordingly and believe that these changes have substantially improved its clarity, methodological transparency, and clinical relevance.

Below, we provide a detailed point-by-point response.

Reviewer#1

Comment 1: Terminology (“fascia”) is imprecise

Reviewer comment:
Concern regarding the use of “fascia” and lack of anatomical precision.

Response:
We thank the reviewer for this important observation. We have revised the terminology throughout the manuscript to improve anatomical accuracy. Specifically, the term “fascia” has been replaced with “linea alba (aponeurotic layer of the abdominal wall)” where appropriate.

In addition, we have introduced a clarifying statement in the Methods section to ensure consistent and precise terminology throughout the manuscript.

Reviewer#2

Comment 1: Why previous meta-analyses did not establish SB as standard

Reviewer comment:
Request to explain why previous meta-analyses have not established the small bites technique as standard of care and how this study differs.

Response:
We appreciate this important point and have expanded the Discussion accordingly.

We now highlight that previous meta-analyses were often limited by:

- restricted inclusion of studies (primarily RCT-only designs with limited sample sizes)
- heterogeneity in methodology and surgical technique
- limited external validity

In contrast, the present analysis integrates both randomized and well-designed comparative non-randomized studies, thereby increasing sample size and reflecting real-world clinical practice more accurately.

We further emphasize that the gap between evidence and clinical adoption is not solely due to insufficient evidence but also reflects implementation barriers such as surgeon preference, training, and institutional factors.

Additionally, we have incorporated a concise statement in the Conclusion to explicitly address how this study advances beyond previous meta-analyses.

Comment 2: General methodological robustness and interpretation

Reviewer comment (summary):
Well-written and methodologically sound, but clarification on added value requested.

Response:
We thank the reviewer for the positive assessment. In response, we have strengthened the manuscript by:

- clarifying the added value of combining randomized and real-world comparative data
- emphasizing external validity and generalizability
- refining the interpretation of results to avoid overstatement

These changes are reflected in both the Discussion and Conclusion sections

Comment 3: Minor revisions / clarity

Response:
We have carefully revised the manuscript to improve clarity, consistency, and readability throughout. All suggested changes have been implemented.

Additional two further corrections in the revised manuscript were included:

Update of Figure 8_ForestPlot_FCT_SUBITE: The number of shown digits has been shortened to improve readability and consistency. This change is cosmetical only and does not effect any of the data or results

Update of Table 4_GRADE_SUBITE: Correction due to partially incomplete transferred data: In the line 'Fascial Dehiscence', one non-RCT study was accidentally not included when extracting the total number of studies and participants, the OR and RR. This created an inconsistency commpared to the correspinding Forest plot in the previous version. This inconcistency is now corrected. The values are updated. The resulting combined OR and RR changed slighlty. The overall conclusion and GRADE assessment remain unchanged.

We are grateful for the reviewers’ insightful comments, which have helped us to improve the manuscript significantly. We believe that the revised version provides a clearer, more robust, and clinically relevant contribution to the field.

We thank you for your consideration.
